# Diagnosis and Management of Mild Traumatic Brain Injury (mTBI): A Comprehensive, Patient-centered Approach

**DOI:** 10.1007/s11916-024-01333-4

**Published:** 2025-01-08

**Authors:** Yevgeniya Sergeyenko, Mollie E. Andreae, Miriam Segal

**Affiliations:** 1 MossRehab Institute for Brain Health, Jefferson Moss-Magee Rehabilitation Hospital, 91 North York Road, Willow Grove, PA USA; 2 Department of Rehabilitation Medicine, Jefferson Moss-Magee Rehabilitation Hospital, Philadelphia, PA USA

**Keywords:** Concussion, Mild traumatic brain injury, Persisting symptoms after concussion, Patient-centered, Rehabilitation, Inter-disciplinary

## Abstract

**Purpose of Review:**

The purpose of this review is to provide an update regarding recent research and recommendations in the care of mild traumatic brain injury (mTBI).

**Recent Findings:**

New diagnostic criteria for mTBI have recently been developed by the American Congress of Rehabilitation Medicine through the Delphi method and this will help to standardize assessment, diagnosis, and treatment.

**Summary:**

Symptoms of mTBI are diverse and can sometimes become persistent. Treatment of mTBI should be patient-centered and may require subspeciality referral and coordinated, inter-disciplinary, or multi-disciplinary treatment.

## Introduction

**Fig. 1:**
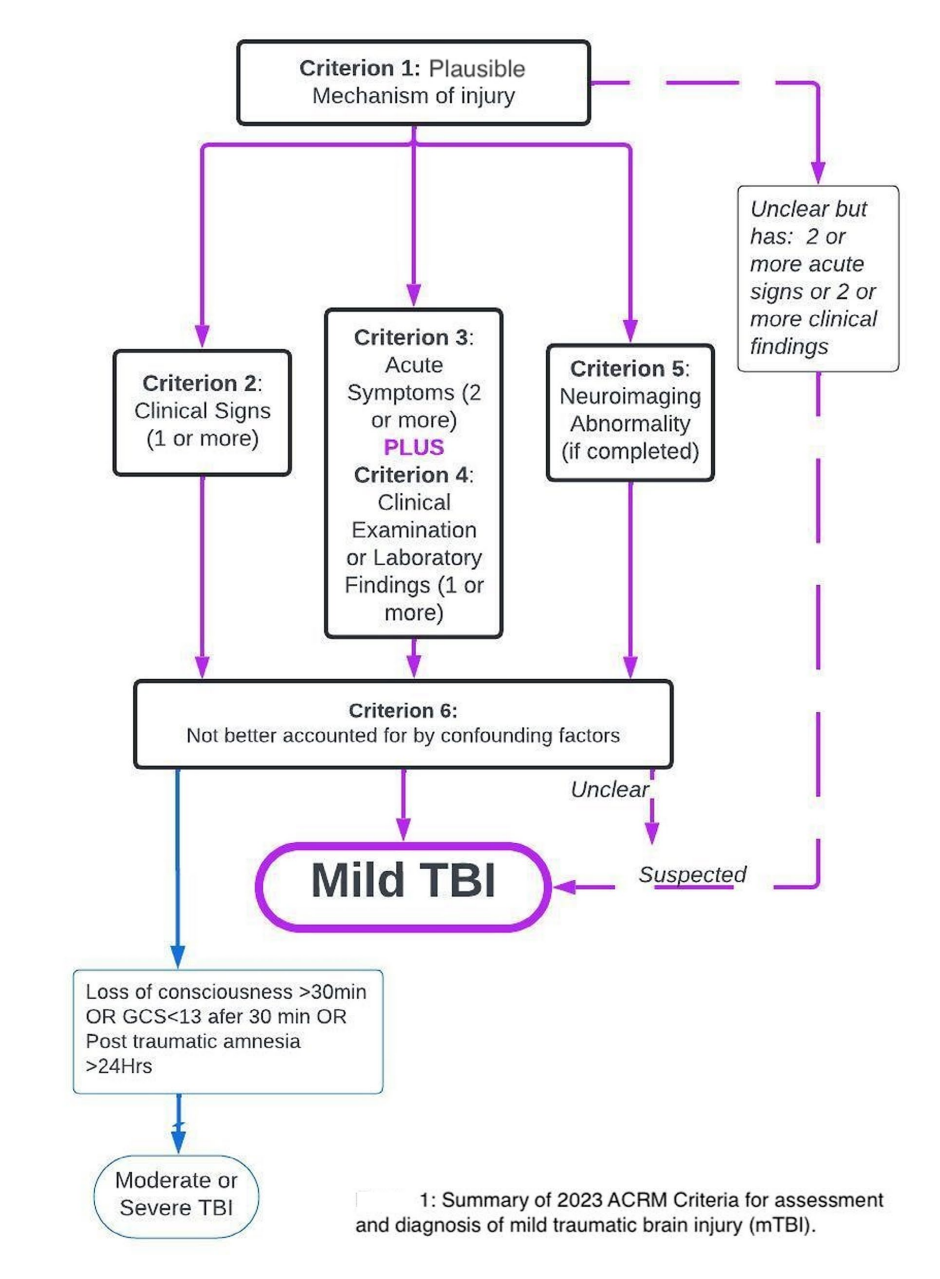
CPHR mTBI Final Paper Sergeyenko Andreae Segal

In general terms, a mild traumatic brain injury (mTBI) results from traumatically induced physiological disruption of brain function, the severity of which does not meet the threshold for moderate or severe traumatic brain injury. This is the broad concept upon which the 1993 American Congress of Rehabilitation Medicine (ACRM) definition of mTBI was based [1]. Since that time, the understanding of mTBI has grown, creating the necessity for a more nuanced definition. Since its publication, the 1993 definition has been succeeded by various other diagnostic criteria, resulting in diagnostic inconsistency in the literature and in the clinic [2, 3]. In 2023, the ACRM published new diagnostic criteria for mTBI, formulated to be appropriate across the lifespan and in sports, civilian trauma, and military settings [2]. These diagnostic criteria for mTBI were developed over four years through the Delphi method, a rigorous process for establishing expert consensus. The reader is encouraged to refer to the published criteria for a detailed understanding, but they are summarized here, as well as in Fig. 1 and Table 1.

**Table 1:** 2023 ACRM Criteria for diagnosis of mTBI: definitions

Criterion 1	AND	Criterion 2	OR	Criterion 3 PLUS Criterion 4		OR	Criterion 5	AND	Criterion 6
Mechanism of Injury ***(Necessary)***		Clinical Signs (1 or more)		Acute Symptoms (2 or more)	Clinical Examination and Laboratory Findings (1 or more)	Neuroimaging ***(Not necessary)***		Not better accounted for by confounding factors. ***(Necessary)***	
Head struck by an object		Loss of consciousness immediately following an injury		Acute subjective alteration in mental status; feeling confused, dazed, disoriented	Cognitive impairment on acute clinical examination	Evidence of TBI on computed tomography (CT)		Pre-existing or co-occurring health conditions Pulmonary-circulatory disruption Syncope prior to a fall	
Head striking a hard object or surface		Alteration of mental status immediately following injury or following regaining consciousness		Physical symptoms: headache, nausea, dizziness, problems with balance, vision problems, photophobia, phonophobia	Balance impairment on acute clinical examination	Evidence of TBI on magnetic resonance imaging (MRI)		Alcohol or substance intoxication or medication use	
Acceleration/deceleration forces acting on the brain		Post traumatic amnesia		Cognitive symptoms: brain fog, memory problems, difficulty concentrating	Oculomotor impairment or symptom provocation in response to vestibular-oculomotor challenge on acute clinical examination			Acute stress reaction to trauma	
Blast or explosion exposure		Other acute neurologic signs such as observed motor incoordination, seizure, or tonic posturing immediately following injury		Emotional symptoms: mood lability, irritability	Elevated blood biomarker(s) indicative of intracranial injury			Symptom exaggeration	

Diagnosis of mTBI begins with obtaining a history of a plausible mechanism of injury (Criterion 1, Fig. 1; Table 1). The mechanism of injury is one that results in a transfer of mechanical energy to the brain from external forces. This occurs from the head being struck with an object, the head striking an object or surface, the brain experiencing acceleration/deceleration forces without direct contact to the head, or experiencing exposure to forces generated from a blast or explosion [2]. After a plausible mechanism is established, one or more of the following conditions must be met: one or more clinical signs attributable to brain injury or at least two acute symptoms together with at least one clinical or laboratory finding attributable to brain injury, or neuroimaging evidence of TBI (Fig. 1; Table 1). The criteria further delineate that the terms “concussion” and mild TBI” may be used interchangeably when neuroimaging is normal or not clinically indicated.

Finally, it must be decided that confounding factors do not fully account for any of the above positive criteria used to make the diagnosis. In order for the TBI to qualify as mild, loss of consciousness should be under 30 min, Glasgow Coma Scale (GCS) should be at least 13, and the duration of post traumatic amnesia should be under 24 h [2]. The ACRM criteria for mTBI also provide guidance regarding diagnostic uncertainty when information is missing or unclear and provide criteria for “suspected” mTBI. This allows for a person with an acute suspected mTBI to be treated as if they had an mTBI, thereby mitigating the risks of a false negative diagnosis while maintaining diagnostic precision.

## Approach to Initial Assessment & Co-occurring and Comorbid Conditions

Initial assessment of mTBI should include a complete history and focused physical exam, with special attention paid to the diagnostic criteria discussed above. Neuroimaging should be obtained if indicated. When assessing a patient with mTBI, it is important to remember that this condition does not exist in a vacuum. By its very definition, mTBI results from a transfer of mechanical energy to the brain from external forces [2]. These same external forces can cause co-occurring injuries, resulting in posttraumatic vestibulo-ocular disorder, and/or posttraumatic cervicogenic disorder [4, 5].

Concussion symptom checklists, such as the Postconcussion Symptom Scale [4, 6] and the Rivermeade Post Concussion Symptoms Questionnaire [7], can be used to quickly and easily screen for common symptoms of mTBI and co-occurring disorders (i.e., headache, noise/light sensitivity, dizziness, balance problems, fatigue, sleep disturbance, visual changes, mood dysregulation, autonomic dysfunction, cognitive difficulties [6]). Patient-reported symptom scales can also help to identify which symptoms are most severe and are of highest priority to the patient, and this can be used to guide treatment.

In addition to symptoms of mTBI and co-occurring injuries, it is also important to consider premorbid and comorbid conditions. The prevalence of depression and anxiety amongst American adults has been rising in recent years [8]. In 2020, 9.2% of adults in the United States reported experiencing an episode of major depression in the previous year [8], and more than 15% of adults reported symptoms of anxiety in 2019 [9]. Posttraumatic stress disorder (PTSD) is often identified in military personnel but can also occur in civilian patients who have experienced a traumatic event. Patients are more likely to develop mood dysregulation (i.e., depression, anxiety, post-traumatic stress) and sleep disturbance after mTBI [10], and a prior psychiatric history increases the risk of developing these symptoms after mTBI. Screening tools such as the Patient Health Questionnaire-9 (PHQ-9) and the Generalized Anxiety Disorder-7 (GAD-7) can help to identify mood symptoms [11].

A physical examination of an individual with suspected mTBI should include examination of the oculomotor, vestibular, and craniocervical systems, in addition to examination for trigger points or tender points in the distribution of the occipital and trigeminal nerves. Abnormal smooth pursuits, repetitive saccades, abnormal vestibular-ocular reflex (VOR), and/or impaired convergence are indicative of posttraumatic oculomotor and vestibular dysfunction [5], and can be assessed with the Vestibular/Ocular Motor Screening (VOMS) tool [12]. Balance testing with tandem gait and tandem stance should be performed to identify any dysfunction, and more in-depth screening can be performed with the Balance Error Scoring System (BESS) test [4]. Cervical muscle tenderness or spasm are commonly present in patients with cervical dysfunction [5], and this is usually related to the mechanism of injury (i.e., whiplash injury sustained in a motor vehicle accident). Therefore, cervical range of motion should be assessed, and the neck and shoulders should be palpated for muscle tenderness and spasm [4]. Exercise testing (i.e., Buffalo Treadmill Test or the Buffalo Concussion Bike Test) [4] can identify exercise intolerance and help to guide return to activity, exercise, or play. The Buffalo Concussion Bike Test should be considered in patients who may be unable to walk safely on a treadmill (i.e., those with musculoskeletal injuries, pain, or significant vestibular symptoms [4]). An evaluation of orthostatic vital signs is also useful, as it can reveal autonomic dysfunction when this is present [4].

### Cognitive Screening

In addition to the physical examination described above, a cognitive screen should be performed to identify any potential difficulties in this area. At the minimum, a cognitive screen should include measures of immediate memory, delayed memory, orientation, and concentration. This can be assessed promptly with a standardized measure, such as the Standardized Assessment of Concussion, the Sport Concussion Assessment Tool 6 [4], or the Montreal Cognitive Assessment [13]. More detailed cognitive testing can be performed with computerized assessments, such as the Concussion Vital Signs or the Immediate Post-concussion Assessment and Cognitive Testing (ImPACT) [4]. For individuals who complain of persistent cognitive difficulties after mTBI, formal neurocognitive testing performed by a neuropsychologist should be strongly considered [4].

### Approach to Initial Treatment/Management

Due to the diversity of the presentation of mTBI symptoms, no one treatment approach or guideline is sufficient to meet the needs of every patient, despite the existence of several guidelines and consensus statements. Reassuringly, evidence has shown that patients who sought early treatment and guidance from a health care provider had quicker recovery and reduced risk of developing persistent symptoms [14, 15]. Patients and their families should be provided with quality, patient-oriented education materials, information regarding red flag symptoms (i.e., severe headache, lethargy, slurred speech, vomiting, and convulsions), and reassurance regarding the usual recovery period of two to four weeks [16]. Clinicians should also consider the importance of positive expectation setting regarding severity and duration of symptoms as it has been shown to result in superior functional outcomes [17, 18].

### Relative Rest

Once a diagnosis of mTBI has been made, regardless of the mechanism of injury, it is recommended that patients undergo a period of relative rest for 24–48 h, followed by a gradual return to physical and cognitive activity [19–21]. Due to a lack of supportive evidence, strict rest, consisting of isolation and complete stimuli avoidance until acute symptom resolution has occurred, is no longer recommended [22, 23]. In fact, several studies found that this approach can result in a higher symptom burden, prolonged recovery period, and an increased risk of developing persistent symptoms when compared to relative rest [16, 20, 24–26]. A case-control study demonstrated that those who underwent strict rest for greater than two days were less likely to have returned to school or work 1–2 months post injury [27]. The purpose of relative rest is to reduce the overall metabolic demand on the brain, not to avoid all activity entirely. During this time, patients should minimize screen time and excess cognitive burden but are encouraged to participate in activities of daily living and light aerobic activity, such as walking, as long as symptom exacerbation remains mild [16, 19]. Physical activities that are high in intensity or ones that increase risk of fall or recurrent head trauma should be avoided, giving special consideration to those individuals with significant vestibular symptoms.

The specifics of cognitive rest have not been as well defined as physical rest, but typically involve avoidance of activities that require attention and concentration. Brown et al. performed a prospective cohort study using a self-reported cognitive activity scale rate 0–4. They found that patients who reported the highest levels of cognitive activity following a concussion took longer to recover [28]. But much like physical activity during the relative rest period, the evidence does not support complete cognitive rest as individuals in lower quartiles of cognitive activity had similar recovery times [28]. Screen time is another important component of cognitive rest to consider. According to a randomized study by Macnow et al., individuals who abstained from screen usage during the first 48 h following a concussion experienced symptom resolution in an average of 3.5 days compared to 8.0 days in those who did not [29]. However, beyond 48 h, there is limited guidance on screen time and its role in the post-concussion recovery process.

### Sub-Symptom Threshold Exercise

After the period of relative rest is over, patients may progress to a gradual return to physical and cognitive activity. Return to physical activity can be done systematically through a sub-symptom threshold exercise program. Patients undergo an initial exercise tolerance evaluation with the Buffalo Concussion Treadmill Test (BCTT). During this evaluation, the threshold hear rate (HR) is identified by increasing exercise intensity until symptom exacerbation occurs. The target sub-symptom HR is then calculated as 80% of the threshold HR [22, 30]. This then serves as the guide for the twenty-minute aerobic exercise prescriptions after mTBI. Individuals progress at different speeds, but the goal is to be able to exercise to exhaustion at 85–90% of age-predicted maximum HR for twenty minutes without symptom exacerbation [22, 30]. Once this milestone has been achieved, a return to sport or more strenuous activity can be considered. A recent meta-analysis [25] demonstrated that prescribed sub-symptom threshold exercise can safely be administered as early as two days post injury and can reduce the incidence of persisting symptoms.

## Return to Life Roles

### Return to Sport

For those patients wishing to return to sport, it is recommended they undergo a return to play progression consisting of six stages with at least 24 h in between the completion of each stage. If mTBI symptoms worsen, they must return to the prior level. The first stage begins with symptom-limited activity (i.e., usual activities of daily living) followed by light aerobic activity (i.e., walking or stationary bike). They then progress to sport-specific exercises (i.e., running, sprinting, sport-specific warmups and drills). Stage four consists of non-contact training drills (i.e., more intense training, weightlifting, and resistance training). Full contact practice is the second to last stage and is meant to restore confidence and allow for full participation, including scrimmaging. The progression ends with the final stage of return to play, which consists of normal game [16]. Depending on the individual, it can take anywhere from one week to greater than one month to complete the progression [16, 19].

### Return to Learn/Return to Work

Returning to an academic setting is approached in a similar stepwise fashion as return to play with the additional component of school accommodations across four domains. Environmental and physical accommodations include modified attendance, frequent rest breaks, a learning environment with minimal disruptions, and avoidance of activities with increased risk of fall or collision. Curriculum and testing accommodations allow for reduced course load, pre-printed class notes, and extra time for completion of assignments and tests. The return to learn process consists of four different stages meant to be approached in a step-wise-fashion [31]. The first stage is part of relative rest, which includes daily activities such as reading and minimal screen time. The second stage consists of school activities, including homework, with the purpose of increasing tolerance of cognitive work. The final two stages progress from return to school part-time to full time, as long as symptoms remain mild [19, 32]. If an individual experiences severe symptoms at any of these stages, complete return to the academic environment can be delayed.

The process for return to work is more variable than return to school as it depends on the nature of employment and the degree of physical and cognitive demands placed on the employee. Accommodations like those mentioned in return to school can be implemented, but according to Gourdea et al. gradual return to work, modified duties, and allowances for medical appointments proved to be the most beneficial to employees [33, 34]. The additional aspect of financial loss and gain that accompanies return to work poses additional challenges for both the healthcare provider and the patient [33].

While there is limited evidence regarding the exact timing of follow up, a randomized control study from Wade et al. demonstrated that patients who received follow up from a physician seven to ten days post injury had less social disability and symptom burden six months post injury [35]. Thus, routine follow up is recommended in patients who suffer mTBI to monitor for persistent symptoms and determine when referrals to specialists are necessary [19].

## Management of Persisting Symptoms

For many patients who sustain a mTBI, symptoms improve rapidly in the first two weeks after injury, often resolving entirely within this time frame [36, 37]. However, there is a subset of individuals with mTBI who have persisting symptoms for months or years after the initial injury event [38]. Some literature refers to this as “post-concussion syndrome” (PCS), while other literature refers to the phenomenon as “persisting symptoms after concussion” (PSaC) [17] or “persistent post-concussive symptoms” (PPCS) [4]. While initially thought to affect a minority of patients, over the last decade, it has become increasingly clear that PSaC are more common than previously believed. Some studies have estimated that approximately 15–20% of patients develop persistent symptoms after a concussion or mTBI [20]. However, in a prospective study of PCS, 59% of patients who had sustained a concussion reported persisting symptoms at 1 month after injury and 38% reported persisting symptoms at 1 year [39]. Similarly, in a recent study of recovery from mild TBI, based on data collected from the TRACK-TBI study [40], reported that of 1453 participants, 53% continued to experience functional limitations 12 months post-injury.

Although it is not entirely clear why some individuals go on to develop persisting symptoms while others do not, there is some agreement that this is likely due to a combination of the biological effects of mTBI, psychosocial factors, premorbid and comorbid psychological factors (i.e., depression, anxiety, post-traumatic stress), and premorbid and comorbid physical factors, such as chronic pain [38]. Additionally, studies have identified risk factors for PCS, including high burden of acute and subacute symptoms after mTBI and female sex [38], as well as older age and motor vehicle accident as the etiology of the mTBI [41].

There is, unfortunately, limited evidence regarding the management of PPCS. Treatment is symptom-directed, but typically consists of graded physical exercise, vestibular rehabilitation, manual treatment of the neck and spine, oculomotor vision rehabilitation, psychological treatment, pharmacologic treatment, and interdisciplinary coordinated rehabilitation care [36]. Treatment is complicated by the presence of numerous symptoms, some of which are inter-related and can exacerbate one another. A recent systematic review examined the effectiveness of multidisciplinary care for patients with PPCS and found that multidisciplinary care using a needs-based approach was more effective in reducing concussion-related symptoms and improving mood and quality of life than usual care; however the authors’ conclusions were limited by the large variability in participant demographics, care delivery, and outcomes of the included studies [42].

We therefore recommend a patient-centered approach in which the treating physician works with the patient to identify and treat symptoms of highest severity and priority to the patient. This can be gleaned from the initial history and physical, as well as from concussion and mood symptom checklists and cognitive behavioral screening. In patients with findings indicative of cervical dysfunction, referral for physical therapy should be initiated as soon as possible [5]. Receptors in the neck and suboccipital region provide feedback to the visual, vestibular, and central nervous systems [43]; thus unaddressed cervical dysfunction can prolong, amplify, or even mimic symptoms of mTBI, such as headache, dizziness, blurred vision, and impaired balance.

Posttraumatic oculomotor and vestibular dysfunction may resolve on its own, however, if these findings and associated symptoms (e.g., double vision, blurred vision, dizziness, balance difficulties, vertigo) are present for more than 3–4 weeks, focused rehabilitation therapy (vestibular physical therapy, vision therapy) should be strongly considered [5]. In cases where cervical dysfunction is identified, it should be addressed prior to vestibular and oculomotor dysfunction, as patients with unaddressed cervical dysfunction may not be able to tolerate the maneuvers involved in vestibular and vision therapy due to exacerbation of symptoms [5]. For patients who have predominant mood-related symptoms on history, or who are reporting exacerbation of pre-existing mood disorders, referral should be made to a behavioral health specialist and/or neuropsychologist.

### Pharmacologic Management

There is growing recognition that the acute symptom burden of mTBI is predictive of recovery and that early treatment may serve to avoid progression to symptom persistence [44, 45]. A recent systematic review of pharmacologic treatments identified only a small number of conclusive studies, none which were sufficiently powered to provide any definitive guidance regarding clinical practices [46]. Nevertheless, post traumatic symptoms are often treated individually based on clinical judgment and priorities of the patient.

### Headache

Currently, no FDA-approved drug exists for the treatment of posttraumatic headache (PTH). A small number of studies have examined repurposing various migraine and tension headache medications for treatment of PTH, but a high quality, meaningful evidence basis for treatment is needed [46]. A headache phenotype-based treatment algorithm has been proposed by Ashina et al., using the available evidence and expert opinion [47]. They recommended NSAIDs as a first-line abortive treatment for both tension-type and migraine PTH phenotypes, followed by aspirin-paracetamol-caffeine (for both phenotypes) or for migraine phenotype, a triptan. For prophylactic treatment of the tension-type phenotype, they recommended amitriptyline, mirtazapine, or venlafaxine, and for migraine phenotype, atenolol, bisoprolol, candesartan, metoprolol, propranolol, or amitriptyline [47]. While a role for onabotulinum toxin A and CGRP antagonists was also acknowledged in this paper, the authors anticipated limited access to these costly drugs without an approved indication. In terms of this, we assert that clinicians should be aware of coexisting disorders, such as migraine, and treat accordingly, using these coexisting diagnoses to expand access to potential helpful therapies. Some evidence to support these treatments specifically for PTH is now emerging. A randomized crossover study of 40 patients with persistent PTH found that onabotulinum toxin-A decreased cumulative number of headaches/week by 43.3% in the treatment group and that the cumulative number of headaches/week increased by 35.1% among those in the placebo group [48].

Another important consideration in the treatment of PTH is the co-occurrence of medication overuse headache (MOH), which is formally defined as a headache occurring on 15 or more days/month in a patient with pre-existing headache, developing because of regular overuse of acute headache medication. For triptans and ergots the limit is 10 or more days/month, and for over-the-counter analgesics, the limit is 15 or more days/month. This medication overuse must have occurred for more than 3 months, and the headache usually resolves after the overuse is stopped but not invariably [49]. For some patients, headaches may increase significantly with even lower exposure to these medications, and for opiates and barbiturates it may take only a small exposure to result in a more chronic picture [50]. It has been suggested that a significant proportion of persistent PTH is caused by MOH, but even when medication overuse is present, detoxification therapy does not always yield improvement [51]. We recommend investigating the possibility of MOH and addressing it whenever present.

### Insomnia

Sleep dysfunction is common after mTBI and this can potentiate other symptoms such as fatigue, cognitive difficulty, and headache. Insomnia is a risk factor for prolonged mTBI recovery [52]. Non-pharmacologic therapy, specifically cognitive behavioral therapy for insomnia (CBTi), is recommended as the first line treatment for chronic insomnia in adults but when ineffective, medications are sometimes warranted [5, 53]. Melatonin and low dose amitriptyline, both well-tolerated, have been studied in this population specifically, with some evidence to support their use [45, 54]. Some authors have also recommended short-term use (≤ 4 weeks) of lorazepam, eszopiclone, and zolpidem to treat acute insomnia after mTBI [5]. Nightmares associated with PTSD, a frequent comorbidity of mTBI, especially in military veterans, are often treated with prazosin [55]. In one observational study of 126 combat veterans with blast-induced mTBI, a combination of sleep hygiene counseling and 9 weeks of oral prazosin (titrated up to 7 mg as tolerated) at bedtime improved sleep, reduced headache severity and frequency and also improved cognitive function, and these benefits were sustained at 6 month follow-up [55]. There is also evidence that low-dose clonidine can be used successfully for the treatment of PTSD in veterans [56]. Although the effect of clonidine on insomnia and PTH has yet to be evaluated, if the patient’s PTSD symptoms are contributing to insomnia and/or PTH, clonidine can be a good choice of treatment agent.

### Depressive Symptoms

Depression is common after mTBI and is an important target for treatment [57, 58]. A 2016 meta-analysis showed that antidepressants after TBI may be associated with a reduction in depressive symptoms [59], while a another meta-analysis in 2019 found no significant benefit of antidepressant over placebo in the treatment of major depressive disorder following TBI [60]. Despite the controversy, many practitioners err on the side of offering antidepressants [58]. A 2021 systematic review looking at mTBI specifically, found three studies, which all reported that treatment with sertraline was associated with reduction in depression but reported conflicting data regarding improvement in cognition and overall symptom burden [45].

### Cognitive Difficulties

While some authors have argued against the use of methylphenidate and amphetamines for mTBI [5], there is a small but encouraging body of evidence to support benefits of methylphenidate for the treatment of cognitive dysfunction, depression, and fatigue after mTBI. It has also been suggested to produce a reduction in overall symptom burden [45, 61]. One follow-up study however, done at 5.5 years, demonstrated that these benefits were lost in patients who had discontinued the drug [62]. Clinicians should also bear in mind the possibility of pre-existing or co-existing attention deficit disorders independent from mTBI, which can certainly interact with other symptoms to affect recovery. Other treatments that have been suggested to be of benefit in this area were amantadine [63], sertraline, guanfacine, galantamine, and donepezil as well as the nutraceutical enzogenol [45].

## Conclusion

Mild traumatic brain injury is a complex condition with wide variability in symptoms and clinical course. The 2023 ACRM diagnostic criteria for mTBI will help to bring about an improvement in the characterization of this condition and aid in the development of more precise methods for assessment and treatment. For now, treatment of mTBI should be a shared decision between the patient and treating clinician, with particular focus on management of symptoms that have a likelihood of amplifying other symptoms (i.e., headache, insomnia, mood). There are currently no FDA-approved medications for the treatment of mTBI, and pharmacologic management of mTBI-related symptoms lacks high-quality evidence. Non-pharmacologic treatment should be multi-modal and symptom-directed, prioritizing the treatment of symptoms that are of most importance to the patient, while accounting for co-morbidities and biopsychosocial factors that may also influence recovery. Patients may require referral for graded physical exercise, vestibular rehabilitation, manual treatment of the neck and spine, oculomotor vision rehabilitation, psychological treatment, pharmacologic treatment, and interdisciplinary coordinated rehabilitation care to optimize recovery from mTBI.

## Data Availability

No datasets were generated or analysed during the current study.
